# Antibiotics modulate neoadjuvant therapy efficiency in patients with breast cancer: a pilot analysis

**DOI:** 10.1038/s41598-021-93428-w

**Published:** 2021-07-07

**Authors:** Xi Zhang, Long Yu, Jiajie Shi, Sainan Li, Shiwei Yang, Wei Gao, Shan Yang, Meng Cheng, Haoqi Wang, Zhanjun Guo, Cuizhi Geng

**Affiliations:** 1 Department of Breast Center, The Fourth Hospital of Hebei Medical University, 169 Tianshan Street, Shijiazhuang, 050000 People’s Republic of China; 2 Department of Anesthesiology, The Fourth Hospital of Hebei Medical University, 169 Tianshan Street, Shijiazhuang, 050000 People’s Republic of China; 3 Department of Glandular Surgery, Hebei Provincial People’s Hospital, 348 Heping West Road, Xinhua District, Shijiazhuang, 050011 People’s Republic of China; 4 Department of Rheumatology and Immunology, The Fourth Hospital of Hebei Medical University, 12 Jiankang Road, Shijiazhuang, 050011 People’s Republic of China

**Keywords:** Breast cancer, Tumour immunology

## Abstract

Mounting evidence suggests that microbiota dysbiosis caused by antibiotic administration is a risk factor for cancer, but few research reports focus on the relationships between antibiotics and chemotherapy efficiency. We evaluated the influence of antibiotic administration on neoadjuvant therapy efficacy in patients with breast cancer (BC) in the present study. BC patients were stratified into two groups: antibiotic-treated and control based on antibiotic administration within 30 days after neoadjuvant therapy initiation. Disease-free survival (DFS) and overall survival (OS) were assessed using the Kaplan–Meier method, and the Cox proportional hazards model was used for multivariate analyses. The pathologic complete response rate of the control group was significantly higher than that of the antibiotic-treated group (29.09% vs. 10.20%, *p* = 0.017). Further univariate analysis with Kaplan–Meier calculations demonstrated that antibiotic administration was strongly linked with both reduced DFS (*p* = 0.04) at significant statistical levels and OS (*p* = 0.088) at borderline statistical levels. Antibiotic administration was identified as a significant independent prognostic factor for DFS [hazard ratio (HR) 3.026, 95%, confidence interval (CI) 1.314–6.969, *p* = 0.009] and OS (HR 2.836, 95% CI 1.016–7.858, *p* = 0.047) by Cox proportional hazards model analysis. Antibiotics that initiated reduced efficiency of chemotherapy were more noticeable in the HER2-positive subgroup for both DFS (HR 5.51, 95% CI 1.77–17.2, *p* = 0.003) and OS (HR 7.0395% CI 1.94–25.53, *p* = 0.003), as well as in the T3-4 subgroup for both DFS (HR 20.36, 95% CI 2.41–172.07, *p* = 0.006) and OS (HR 13.45, 95% CI 1.39–130.08, *p* = 0.025) by stratified analysis. Antibiotic administration might be associated with reduced efficacy of neoadjuvant therapy and poor prognosis in BC patients. As a preliminary study, our research made preparations for further understanding and large-scale analyses of the impact of antibiotics on the efficacy of neoadjuvant therapy.

## Introduction

The latest cancer data in 2020 indicates that breast cancer is the most prevalent type of cancer worldwide. In China, breast cancer is the most common malignant tumor and the fifth leading cause of female cancer mortality^1^. The treatment of breast cancer has entered the era of individualized treatment with increasing systemic treatment, especially neoadjuvant therapy—preoperative systemic therapy for breast cancer without distant metastasis. The application of neoadjuvant therapy reduces the tumor burden and increases the likelihood of breast conservation to improve BC patients' quality of life^2^. It also enables clinicians to obtain individual drug sensitivity information on BC patients to guide further treatment^3^. The phenomenon of pathological complete response (pCR) was defined as no residual invasive tumor on pathologic assessment after therapy showing an associated survival benefit^4^.

Antibiotics are required to prevent and treat infectious bacterial diseases, mainly in BC patients with febrile neutropenia (FN), which is a severe adverse effect induced by chemotherapy^5^. Antibiotics are effective for treating various infections. Nevertheless, their application cannot be located as precisely as targeted drugs, which in turn leads to intestinal dysbacteriosis. The gut microbiota participates in many aspects of the human physiological process, from producing nutrients and vitamins to fighting pathogens and protecting immune system development and epithelial mucosa homeostasis^6^. Accumulating evidence suggests that gut microbiota has an impact on the efficacy of anti-tumor therapies—including chemotherapy, immunotherapy, radiotherapy, and surgery—used to treat solid tumors (melanoma, lung cancer, and colon cancer) with numerous mechanisms, including xenometabolism, immune microenvironment, and changed microbial community structure^6^. Antibiotic-treated mice displayed oxaliplatin (OXA) chemoresistance for colon carcinoma and lymphoma compared with specific pathogen-free (SPF) mice, which suggested that antibiotic exposure was associated with reduced chemotherapy efficacy^7^. Further research is needed in breast cancer to study the relationship between the effect of neoadjuvant therapy and antibiotics.

In the present study, we aimed to evaluate the influence of antibiotic administration on neoadjuvant therapy efficacy and prognosis in BC patients. We expect that our findings will provide a basis for future therapeutic concepts in BC patients who require antibiotics during neoadjuvant therapy.

## Method

### Patients

Patients with BC who received new adjuvant chemotherapy followed by surgery at the Fourth Hospital of Hebei Medical University between January 2013 and September 2015 were enrolled in this retrospective study. All methods were performed in accordance with the relevant guidelines and regulations for human participants and were supervised and approved by the Ethics Committee of the Fourth Hospital of Hebei Medical University (No. 2020KS001). Written informed consent was provided by participants. Neoadjuvant therapy consisted of docetaxel (T), anthracycline (A), cyclophosphamide (C), and Herceptin (H), and the types of neoadjuvant therapy were balanced between the two groups (*p* = 0.924, Table S2). All the patients in this study received modified radical mastectomies. According to NCCN guidelines, patients received standard postoperative radiotherapy and endocrine therapy based on postoperative pathological results. In total, 120 BC patients were enrolled in this study, all were of Han ethnicity and from Hebei Province (Table 1). The medical records of all patients were reviewed to determine whether any antibiotic administration occurred within 30 days after neoadjuvant therapy initiation. Data on the specific time of antibiotic exposure, antibiotic class, indication, route of administration, and duration were collected (Table S1). Patients who received antibiotics within 30 days after neoadjuvant therapy initiation were assigned to the antibiotic-treated group, while patients who did not receive antibiotics within 30 days after neoadjuvant therapy initiation were placed in the control group. One of the common reasons why patients use antibiotics is febrile neutropenia (FN) caused by the first cycle of neoadjuvant therapy. Data on additional parameters, including age, primary tumor size, regional lymph node, hormone receptor, HER2 status, and Miller-Payne grading criteria (Mp), were also collected. All patients were followed up every three months until death or until the database was closed (2020-10-24). We ordered the postoperative pathological data of the two groups, evaluated the tumor with Mp grade, and evaluated the lymph node status^8^. We defined pCR as no residual invasive tumor in either the tumor bed or lymph node on pathologic assessment after therapy.

**Table 1:** Clinical characteristics of patients.

Characteristics	Total	ATB-treatment group	Control group	*p*
	n = 120(%)	n = 55 (%)	n = 65 (%)	
**Age**				
≤ 35 years	16 (13.33)	9 (16.36)	7 (10.77)	0.246
35–60 years	73 (60.83)	29 (52.73)	44 (67.69)	
≥ 60 years	31 (25.84)	17 (30.91)	14 (21.54)	
**Primary tumor size**				
Tx	5 (4.17)	1 (1.82)	4 (6.15)	0.206
T1	22 (18.33)	14 (25.45)	8 (12.31)	
T2	70 (58.33)	31 (56.36)	39 (60.00)	
T3	18 (15.00)	6 (10.91)	12 (18.46)	
T4	5 (4.17)	3 (5.46)	2 (3.08)	
**Regional lymph node**				
N0	12 (10.00)	9 (16.36)	3 (4.62)	0.184
N1	34 (28.34)	15 (27.27)	19 (29.23)	
N2	31 (25.83)	14 (25.46)	17 (26.15)	
N3	43 (35.83)	17 (30.91)	26 (40.00)	
**Hormone receptor**				
Positive	82 (68.33)	35 (63.64)	47 (72.31)	0.309
Negative	38 (31.67)	20 (36.36)	18 (27.69)	
**Her-2 status**				
Positive	46 (38.33)	22 (40.00)	24 (36.92)	0.730
Negative	74 (61.67)	33 (60.00)	41 (63.08)	
**Miller-Payne grading system**				
1	16 (13.33)	11 (20.00)	5 (7.69)	0.033
2	27 (22.50)	15 (27.27)	12 (18.46)	
3	28 (23.33)	15 (29.27)	13 (20.00)	
4	23 (19.17)	8 (14.55)	15 (23.08)	
5	21 (17.50)	5 (9.09)	16 (24.62)	
Unable to access	5 (4.17)	1 (1.82)	4 (6.15)	

Some patients' primary tumor cannot be assessed(Tx), so the Mp grading system is not used.

### Statistical analysis

Clinical and pathological features, Miller-Payne grading criteria, and pCR rate were compared by using the chi-square test or fisher’s exact test between two groups. The meaning of disease-free survival (DFS) was the time from operation to the date of disease progression or death. Overall survival (OS) was defined as the time from neoadjuvant therapy initiation to the date of death. At the end of follow-up, 3 (2.5%) patients and 9 (7.5%) patients were lost to follow-up, separately in DFS and OS analysis. We used the Kaplan–Meier method to plot survival curves and the log-rank test to evaluate the prognosis of patients. Univariate and multivariate analyses were done using the Cox proportional hazards model to identify the risk factors for survival. We did statistical analyses by using RStudio, version 1.3.1093 (2009–2020 RStudio, PBC) and SPSS software, version 19.0 (IBM Corporation, Armonk, NY), and *p* < 0.05 was considered statistically significant.

## Result

**Figure 1:**
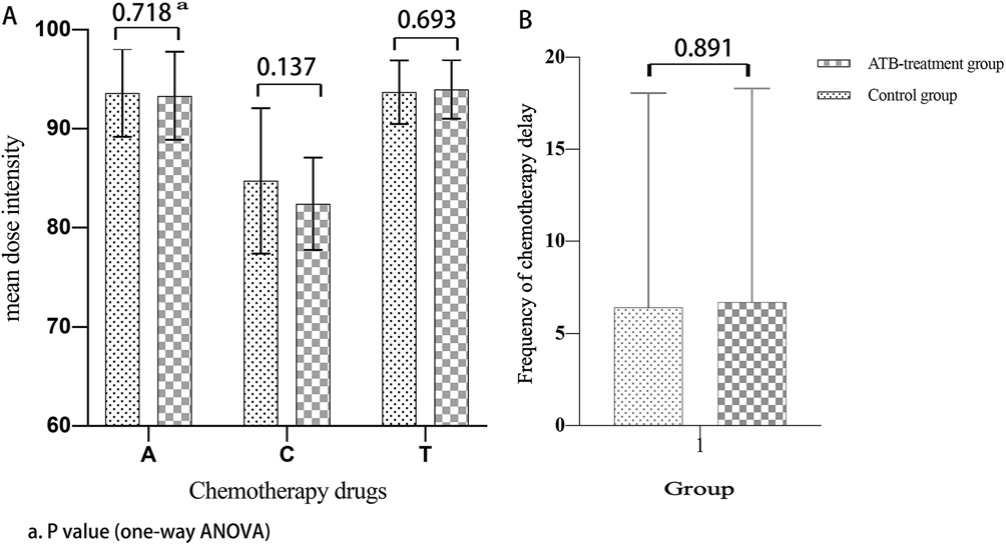
Comparison of dose intensity and frequency of dose delay. (**A**) The mean dose intensity. (**B**) frequency of dose delay.

A total of 120 BC patients who received neoadjuvant therapy were enrolled in this study. The distribution of clinicopathologic characteristics was well balanced between the control and antibiotic-treated groups (Table 1). In the antibiotic-treated group, 15 patients had febrile neutropenia, while the control group had none. There were 16 patients in the antibiotic-treated group who received a reduced dose and 19 patients in the control group (*p* = 0.987). Dose intensity (actual dose received/planned treatment received) and frequency of dose delay (actual delayed number of cycles and planned number of cycles) were not significantly different between the two groups (Fig. 1).

**Figure 2:**
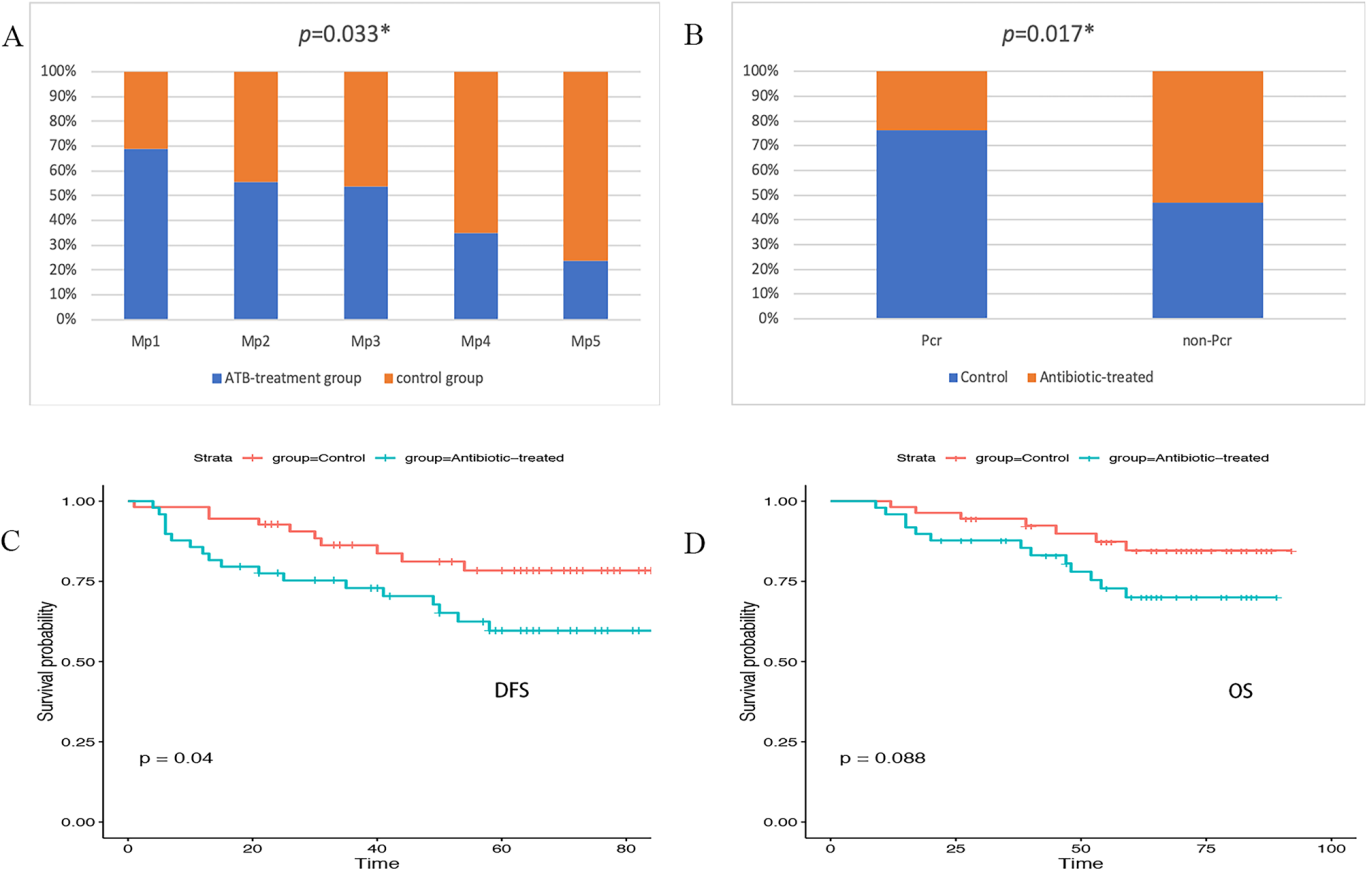
The impact of antibiotic administration on the clinical outcome and efficacy of BC patients. (**A**) Miller-Payne grade in BC patients. (**B**) Analysis of the pathological complete response (pCR) rate in BC patients. (**C**) The Kaplan–Meier curve of progression-free survival (DFS). (**D**) The Kaplan–Meier curve of overall survival (OS).

The effect of antibiotic administration on the efficacy of neoadjuvant therapy was evaluated in BC patients. We collected postoperative pathological information estimated using the Miller-Payne grading system. As shown in Fig. 2A, the distribution frequency of Miller-Payne grades was significantly different between these two groups (*p* = 0.033), with the proportion of Miller-Payne grades 2 and 3 increasing in the antibiotic-treated group and Miller-Payne grades 4 and 5 increasing in the control group (Fig. 2A). The pathological complete response (pCR) rate was also significantly higher in the control group than in the antibiotic-treated group (29.09% vs. 10.20%, *p* = 0.017) (Fig. 2B). These data demonstrated that antibiotic-induced microbiota dysbiosis might decrease chemotherapy treatment efficiency, referring to the Miller-Payne grading system in BC patients.

Further univariate analysis with Kaplan–Meier calculations demonstrated a significant difference in DFS (*p* = 0.04) and a borderline significant difference in OS (*p* = 0.088) between antibiotic-treated and control BC patients (Fig. 2C,D).

The potential outcome predictors were analyzed using the Cox proportional hazards model for multivariate analysis. As shown in Tables 2 and 3, antibiotic administration was identified as a significant independent prognostic factor for DFS (HR 3.026, 95% CI 1.314–6.969, *p* = 0.009) and OS (HR 2.836, 95% CI 1.016–7.858, *p* = 0.047). Furthermore, HER2 status was also identified as a significant independent prognostic factor for DFS (HR 2.946, 95% CI 1.306–6.645 *p* = 0.009) and OS (HR 6.320, 95% CI 2.235–17.872, *p* < 0.001). These data demonstrated that antibiotic-induced microbiota dysbiosis might modify BC patient outcomes by decreasing chemotherapy treatment efficiency.

**Table 2:** Univariate and multivariate analyses of DFS in BC patients.

Prognostic Factor	Univariate analysis	*p*-value	Multivariate analysis	*p*-value
	DFS		DFS	
	HR (95%CI)		HR (95%CI)	
ATB/Control	2.2 01 (0.454–4.770)	0.045*	3.026 (1.314–6.969)	0.009**
Age				
≤ 35 year/35-60 year/ ≥ 60 year	1.772 (0.801–3.917)	0.158	1.633 (0.711–3.750)	0.247
Primary tumor size				
T1/T2/T3/T4	1.530 (0.650–3.601)	0.330	1.761 (0.714–4.337)	0.219
Regional lymph node				
N0/N1/N2/N3	1.412 (0.638–3.127)	0.395	1.703 (0.750–3.864)	0.203
Hormone receptor				
Positive/negative	0.822 (0.372–1.818)	0.629	1.077 (0.468–2.480)	0.863
Her-2 status				
Positive/negative	2.509 (1.180–5.335)	0.017*	2.946 (1.306–6.645)	0.009**
Miller-Payne grading system				
Mp1/Mp2/Mp3/Mp4/Mp5	0.862 (0.517–1.437)	0.568	1.139 (0.479–2.709)	0.769
Pathologic complete response				
Non-pCR/ pCR	0.618 (0.214–1.781)	0.373	0.720 (0.144–3.608)	0.689

*BC* breast cancer, *DFS* disease-free survival, *ATB* antibiotics;

Significant. codes: 0 ‘**’ 0.01 ‘*’ 0.05 ‘.’ 0.1 ‘ ’ 1.

**Table 3:** Univariate and multivariate analyses of OS in BC patients.

prognostic factor	Univariate analysis	*p*-value	Multivariate analysis	*p*-value
	OS		OS	
	HR (95%CI)		HR (95%CI)	
ATB/Control	2.179 (0.869–5.463)	0.097	2.836 (1.016–7.858)	0.047*
Age				
≤ 35 year /35-60 year / ≥ 60 year	2.380 (0.972–5.826)	0.058	2.012 (0.769–5.262)	0.154
Primary tumor size				
T1/T2/T3/T4	1.436 (0.522–3.953)	0.483	1.737 (0.585–5.159)	0.320
Regional lymph node				
N0/N1/N2/N3	0.981 (0.400–2.410)	0.967	1.389 (0.512–3.839)	0.502
Hormone receptor				
Positive/negative	0.944 (0.363–2.459)	0.907	1.403 (0.512–3.839)	0.510
Her-2 status				
Positive/negative	4.614 (1.759–12.1)	0.0018**	6.320 (2.235–17.872)	< 0.001***
Miller-Payne grading system				
Mp1/Mp2/Mp3/Mp4/Mp5	0.656 (0.348–1.237)	0.193	0.739 (0.275–1.989)	0.549
Pathologic complete response				
Non-pCR/pCR	0.416 (0.097–1.793)	0.239	0.878 (0.110–7.010)	0.903

*BC* breast cancer, *OS* overall survival, *ATB* antibiotics;

Significant. codes: 0 ‘***’ 0.001 ‘**’ 0.01 ‘*’ 0.05 ‘.’ 0.1 ‘ ’ 1.

**Figure 3:**
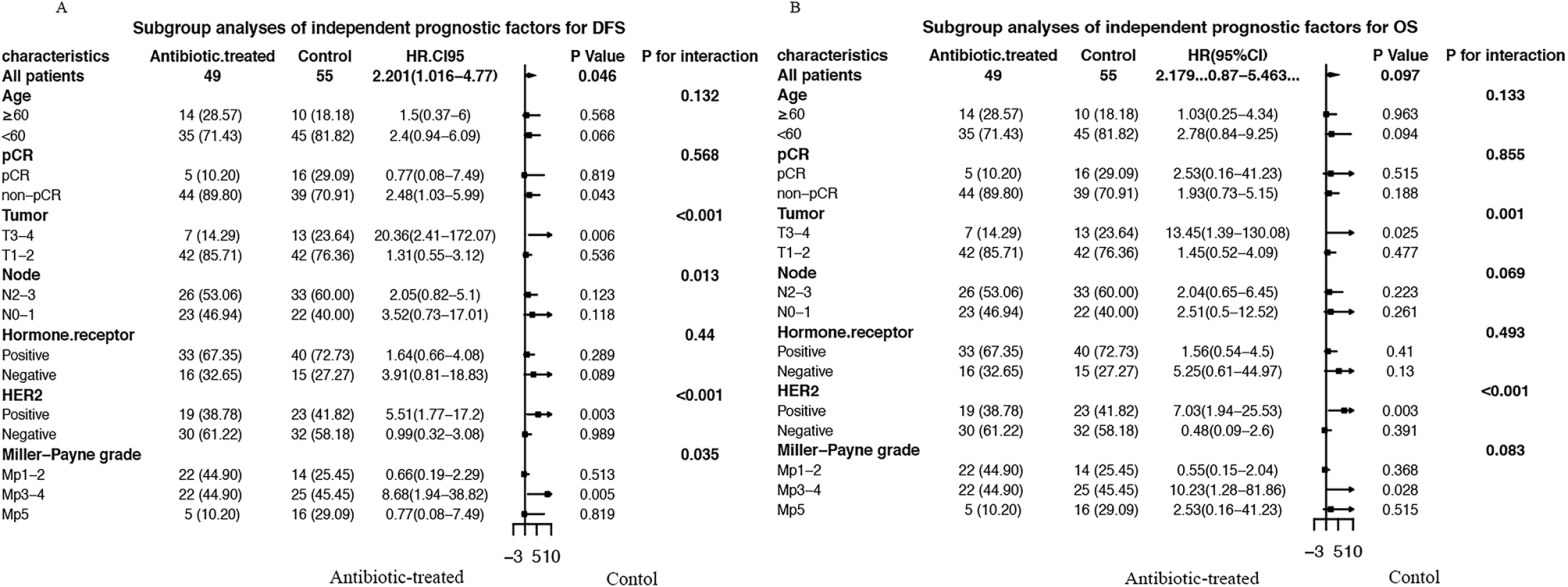
Subgroup analyses of independent prognostic factors for DFS (**A**) and OS (**B**).

The influence of antibiotic administration on DFS and OS was further investigated within individual subgroups of BC patients by stratified analysis, and the antibiotic-treated group displayed a trend of reduced DFS (Fig. 3A) and OS (Fig. 3B) within most subgroups. Antibiotics that initiated reduced efficiency of chemotherapy were more noticeable in the HER2-positive subgroup for both DFS (HR 5.51, 95% CI 1.77–17.2, *p* = 0.003) and OS (HR 7.03, 95% CI 1.94–25.53, *p* = 0.003), as well as in the T3-4 subgroup for both DFS (HR 20.36, 95% CI 2.41–172.07, *p* = 0.006) and OS (HR 13.45, 95% CI 1.39–130.08, *p* = 0.025) by stratified analysis. The *p*-values for interaction were less than 0.05 in both the HER2 subgroup and T3-4 subgroup, indicating that antibiotic administration might change the outcome in HER2-positive BC patients and those whose primary tumor was larger than 5 cm.

## Discussion

We estimated the effect of antibiotic administration on neoadjuvant therapy and prognosis in BC patients. We found that patients who administrated antibiotics might have a decreased treatment efficiency of neoadjuvant therapy referring to the Miller-Payne system and ORR. It is known that the composition of the intestinal flora of cancer patients is different from that of individuals without cancer. The intestinal flora plays an important role in the human immune system^9,10^. Antibiotics induce dysbacteriosis by changing the intestinal flora environment involved in tumorigenesis, causing inflammation and disorders of the immune system^11,12^. The gastrointestinal microbiota could influence cancer immunotherapy on both the prognostic and therapeutic sides^13^, and the application of antibiotics could reduce the clinical benefit of immune checkpoint inhibitors (ICIs) in renal cell carcinoma (RCC) and non-small-cell lung cancer (NSCLC)^14^. A previous article also found that antibiotics could affect the efficiency of chemotherapy in esophageal cancer^15^. Consistent with a previous report, we discovered that antibiotic-induced dysbacteriosis might also modified neoadjuvant therapy efficiency in BC patients.

Changes in the microbial community structure may affect the efficacy of anti-tumor therapy, including chemotherapy. Intestinal dysbacteriosis reduces regulatory T cells (Tregs) and increases Th1 and Th17 cells to modulate the immune microenvironment of tumors, resulting in cyclophosphamide (CTX) chemoresistance^7^. Similar results were observed with oxaliplatin (OXA) treatment^7^. In addition to immune modulations, bacterial translocation can also directly modulate chemotherapy efficacy. In colorectal cell lines (HCT116 and HT29), autophagy of *Fusobacterium nucleatum* was associated with chemoresistance to 5-fluorouracil (5-FU) and oxaliplatin (OXA)^16^.

A recent study showed that gut microbiota was directly involved in the immune-mediated trastuzumab anti-tumor efficacy in 24 BC patients and mice^17^. Researchers used antibiotic administration or fecal microbiota transplantation from antibiotic-treated donors to change the community structure of gut microbiota to influence treatment efficiency^17^. Fecal microbiota transplantation provided an opportunity to correct antibiotic-induced chemotherapy failure. Our study was retrospective and lacked information on the intestinal flora of patients at the beginning of neoadjuvant therapy. It is vital to identify the gut microbiota responsible for dysbacteriosis-related treatment inefficiency in BC patients. The discovery of targeted bacteria capable of rescuing antibiotic-associated dysbiosis made it feasible to promote anti-tumor efficacy by modulating microbiome diversity, especially for HER2-positive BC patients.

In our study, the pathological complete response (pCR) rate was significantly higher in the control group than in the antibiotic-treated group (29.09% vs. 10.20%, *p* = 0.017). However, the pCR rate was not a significant independent prognostic factor for DFS and OS. The pCR rate is an effective indicator of short-term treatment in neoadjuvant therapy^4^. However, most studies show that the pCR rate is higher in hormone receptor (HR)-negative breast cancer patients after neoadjuvant therapy and improves DFS and OS outcomes compared patients with less than a pCR. However, there was no advantage in overall survival compared with HR-positive patients^18^. As this study was small and there were 82 (68.33%) HR-positive patients in our study, the pCR rate was not a significant factor for determining DFS or OS.

HER2 status was a significant independent prognostic factor of breast cancer, and we obtained the same conclusion in our study. In the subgroup analysis, the use of antibiotics dramatically decreased the treatment efficiency in HER2-positive and T3-4 subgroups of BC patients. Cancer patients may be treated with antibiotics because of conditions such as chemotherapy-related agranulocytosis, malnutrition, and cachexia^19^. We hope that our research draws clinicians’ attention to optimizing management of cancer patients during chemotherapy to reduce the incidence of chemotherapy-related side effects. As this study was small and retrospective, no conclusion for clinical practice is possible. More trials are necessary to assess the benefits and risks of antibiotics in breast cancer patients receiving neoadjuvant therapy.

## Conclusion

Antibiotic administration might be associated with reduced chemotherapy efficacy and poor prognosis in BC patients, especially in HER2-positive BC patients and patients whose primary tumor was larger than 5 cm. As a preliminary study, our research made preparations for further understanding and large-scale analyses of the impact of antibiotics on the efficacy of neoadjuvant therapy.

## Supplementary Information

Supplementary Information.

## Data Availability

All data generated or analyzed during this study are included in this published article (for information, please contact the corresponding author). Ethics Approval and Informed Consent was obtained.
