# Supplementary material for: Antibiotics modulate neoadjuvant therapy efficiency in patients with breast cancer: a pilot analysis
Source: Sci Rep. 2021 Jul 7;11:14024. doi: 10.1038/s41598-021-93428-w (PMC8263554; doi:10.1038/s41598-021-93428-w)
Supplement: Supplementary file 1 — Supplementary Information. [file 41598_2021_93428_MOESM1_ESM.pdf]

Antibiotics Modulate Neoadjuvant Therapy Efficiency in Patients with Breast Cancer – a Pilot Analysis

Xi Zhang[1], Long Yu[2], Jiajie Shi[1], Sainan Li[1], Shiwei Yang[3], Wei Gao[1], Shan Yang[1], Meng Cheng[1], Haoqi Wang[1], Zhanjun Guo[4], Cuizhi Geng[1]*.

1. Department of Breast Center, The Fourth Hospital of Hebei Medical University, 169 Tianshan Street, Shijiazhuang 050000, PR China.
2. Department of Anesthesiology, The Fourth Hospital of Hebei Medical University, 169 Tianshan Street, Shijiazhuang 050000, PR China.
3. Department of Glandular Surgery, Hebei Provincial People's Hospital, 348 Heping West Road, Xinhua District, Shijiazhuang 050011, PR China.
4. Department of Rheumatology and Immunology, The Fourth Hospital of Hebei Medical University, 12 Jiankang Road, Shijiazhuang 050011, PR China.

* Corresponding author:

Cuizhi Geng

Ph.D. and M.D.

Department of Breast Center, The Fourth Hospital of Hebei Medical University, 169 Tianshan, Street, Shijiazhuang 050000, PR China.

E-mail: bsfxwz@163.com

Table S1: The statues of ATB administration.

		ATB-treatment group(n=48)
Type of ATB	β-lactams	43
	Quinolones	12
Duration	＜7days	55
	≥7days	0
ATB-administration	Intramuscular/Intravenous	55
	Oral	0
ATB indication	Febrile neutropenia (FN)	55

ATB: antibiotics.

Table S2: The Neoadjuvant therapy of BC patients

a. docetaxel(T)，anthracycline（A）， cyclophosphamide (C) and herceptin (H).

Neoadjuvant therapy	ATB-treatment group	control group	P Value
TAC/TEC	8	13	0.924
TA/TE	26	23	
AC/EC-T	11	18	
AC/EC-TH	1	1	
AC	9	9	
TCH	0	1	
